# The role of lactate metabolism in shaping intestinal microbiota and health

**DOI:** 10.3934/microbiol.2026020

**Published:** 2026-07-23

**Authors:** Carlos Sabater, Xenia Vázquez, Sylvia H. Duncan

**Affiliations:** 1 Rowett Institute, University of Aberdeen, Foresterhill, Aberdeen, Scotland, AB25 2ZD, UK; 2 Group of Functionality and Ecology of Beneficial Microorganisms (MicroHealth), Dairy Research Institute of Asturias (IPLA-CSIC), Francisco Pintado Fe nº 26, 33011, Oviedo, Asturias, Spain; 3 Health Research Institute of Asturias (ISPA), Avenida Hospital Universitario s/n, 33011 Oviedo, Asturias, Spain

**Keywords:** lactate, probiotics, butyrate, propionate, *Anaerobutyricum*, microbial metabolism, gut health

## Abstract

Lactate, particularly the D-isomer, which is produced exclusively by microbial fermentation, is usually low or undetectable in the healthy colon but can accumulate when there is a microbial imbalance, contributing to low pH and inflammation. Intestinal lactate-utilizing bacteria, such as *Anaerobutyricum soehngenii*, *Anaerostipes* species, and *Coprococcus catus*, convert lactate to short-chain fatty acids, including butyrate, potentially important for health. Here, we mined genomes of lactate utilizers for genes involved in propionate formation and conducted metabolic network modeling to investigate the cross-feeding mechanism. Genomes were also mined for antibiotic resistance genes, important when considering strains for biotherapeutics. Metabolic models were computed to elucidate the potential cross-feeding mechanisms between lactate utilizers in the human gut. Bioinformatics analysis revealed that the butyryl CoA:acetate CoA-transferase route was the dominant pathway for butyrate formation. The propanediol utilization compartment was annotated in *A. halli*, *A. soehngenii*, *Eubacterium limosum*, and *C. catus*. Of the genomes of key lactate utilizers assessed for antibiotic resistance genes, several possessed genes for multidrug resistance; however, other strains of *Anaerostipes*, *C. catus*, and *Megasphaera elsdenii* exhibited no antibiotic resistance genes. Metabolic modeling revealed that lactate-utilizing strains, including *Anaerotignum*, *Eubacterium*, and *Anaerostipes*, are key symbionts in maximizing the metabolic capabilities of the gut microbial community. Clinical interest in lactate-utilizers as next-generation probiotics is developing, and these data underscore the exciting therapeutic potential of targeting lactate metabolism to restore microbial and metabolic balance, in addition to potentially mitigating inflammatory diseases.

## 1. Introduction

The remarkable complexity and functionality of the human intestinal microbiota are recognized as key drivers of host health [1],[2]. The human large intestine is comprised of hundreds of different bacterial species, the majority of which are anaerobes that ferment both dietary substrates that escape digestion by host enzymes, including alpha amylase, and host components, such as mucin, to short-chain fatty acids (SCFAs) [3]–[5], notably acetate, propionate, and butyrate [5],[6]. These metabolites are generally considered beneficial for health and have intricate signaling capacities, resulting in improved intestinal barrier function [7]. Several short-chain fatty acids, such as formate, acetate, and lactate, can be used by cross-feeding bacteria. For example, formate is produced by many bacterial species but is usually only detected at low levels due to utilization of the product by cross-feeding bacteria such as *Blautia hydrogenotrophica* that can use formate to generate acetate via the Wood Ljungdahl pathway [8]. Acetate is usually the highest SCFA in the colon, being used to fuel bacteria employing the butyryl CoA:acetate CoA route for butyrate formation [9], which is the dominant route for butyrate formation in the human colon despite being energetically less favorable than the butyrate kinase route [10]. This is likely to be due to the constant supply of acetate in the colon, as this is a fermentation product of many colonic bacterial species.

In the healthy colon, another microbial fermentation product, lactate, is generally only found in low levels or may be undetectable. However, in certain bowel conditions, such as short bowel syndrome and inflammatory bowel disease, lactate levels, particularly D-lactate, which is not produced by human tissuehave been found to be much higher [11]. Elevated D-lactate may therefore serve as a biomarker for disease and microbial imbalance (Figure 1). Lactate has a lower acid dissociation constant (pKa) than the other main fermentation acids detected in the colon, and the accumulation of lactate can lower the colonic pH, resulting in potential changes in the composition of the gut microbiota [12], especially pH-sensitive microbes such as *Faecalibacterium* species [13], and causing damage to the colonic mucosa and exacerbating inflammation. Moreover, these changes can result in the overgrowth of lactate producers and a loss of lactate-utilizing species, in particular those that are pH sensitive [13],[14]. Moreover, certain lactate-utilizing bacterial species, such as *Desulfovibrio piger*, can also generate hydrogen sulfide, which can inhibit butyrate oxidation and damage the epithelial barrier [15],[16].

There is clearly a good understanding of the bacteria and enzyme systems that are used by gut bacteria to ferment dietary carbohydrates [5]. The metabolic routes for SCFA formation by dominant gut bacteria are also emerging [17]. However, their metabolic plasticity is less well understood, which is important when considering the development of next-generation probiotics along with other factors such as antibiotic resistance genes.

**Figure 1.**
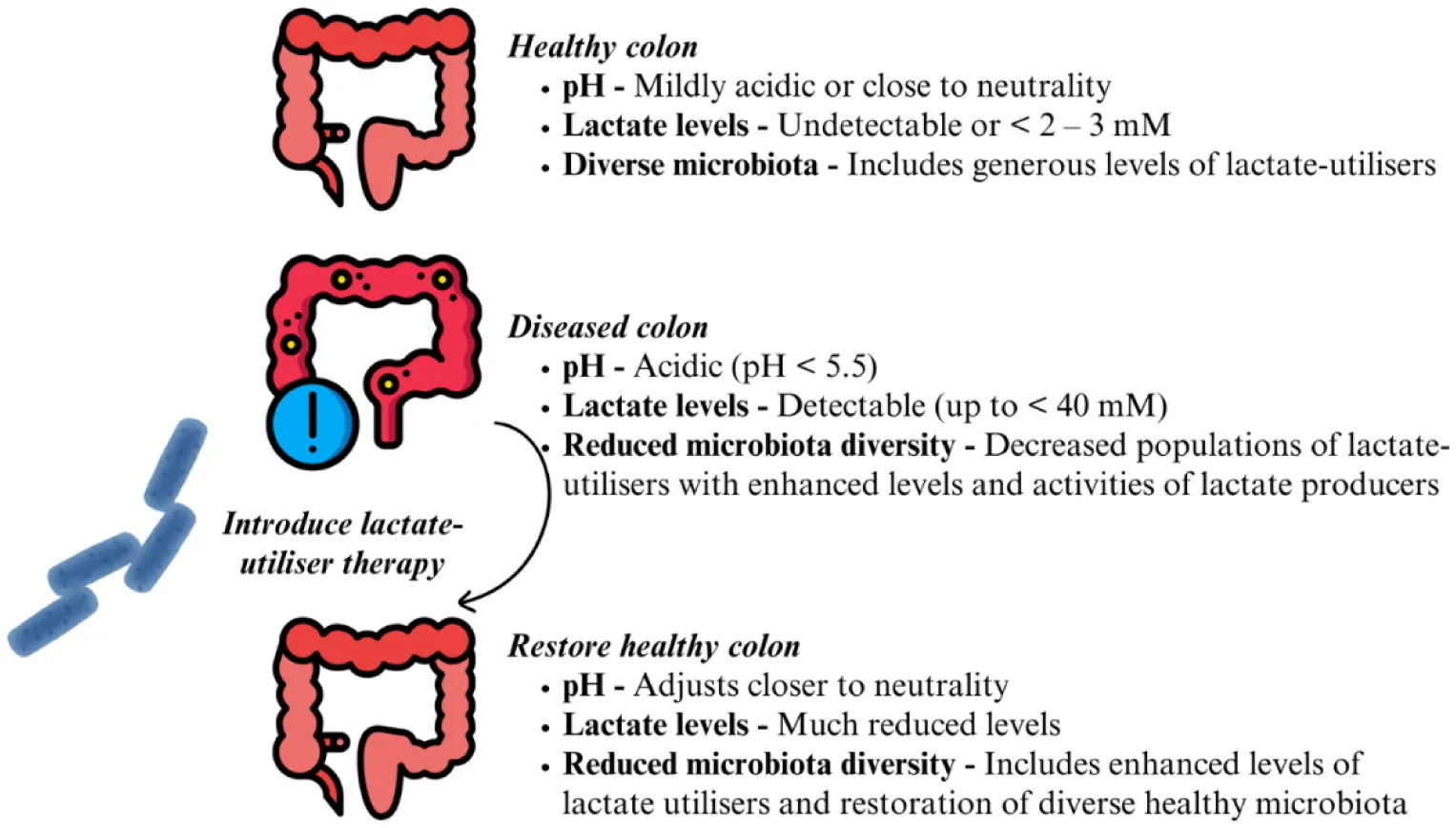
Schematic indicating the impact of lactate accumulation in the colon and the role of lactate bacterial-utilizer therapy to metabolize and reduce total lactate levels and to restore gut health. This figure has been designed using resources from flaticon.com (free icons “colon” by iconixar and “intestines” by SMashicons).

## 2. Lactate-producing bacteria from the human colon

Many bacteria in the large intestine possess the ability to produce lactate as a fermentation product from the metabolism of carbohydrates, usually in the form of oligosaccharides and soluble sugars [11]. Lactate-producing bacteria are relatively more abundant in early life, creating an acidic environment that suppresses harmful bacterial species. The prevalence of lactate-producing species is lower in healthy adults, as a myriad of bacterial species colonize the colon [18]. Key lactate-producing bacteria include *Lactobacillus* species, which mainly produce L-lactate by glycolysis and tend to be more prevalent in the small intestine [11]. *Bifidobacterium* species, several of which can metabolize milk oligosaccharides, can also contribute to the total lactate pool following the metabolism of carbohydrates employing the bifid shunt pathway, which results in the formation of mostly acetate and lactate [19]. The dominant *Bifidobacterium* species in infants include *B. longum*, *B. breve*, and *B. bifidum*, whilst in adults, *B. adolescentis* tends to be more prominent. *Bifidobacterium* species generally produce L-lactate, although some strains can produce small amounts of D-lactate. Bacterial species belonging to the *Bifidobacterium* genus generate lactate by employing the bifid shunt (fructose-6-phosphate phosphoketolase) pathway. The exact stereochemistry can vary by species and strain. Further bacterial species generating lactate include *Clostridium* and *Prevotella* species, but not usually as a major fermentation product. Other major lactate producers belong to the *Streptococcus* and *Enterococcus* genera. These also employ the glycolysis route for lactate formation but are not usually dominant in healthy colons, being considered opportunistic pathogens [20]. *Streptococcus* species can produce both stereoisomers. Lactate-producing bacteria can also possess the enzyme lactate racemase, which catalyzes the reversible interconversion of the two lactate stereoisomers.

Lactate, therefore, exists as two stereoisomers, L-lactate and D-lactate. L-lactate is the main isomer formed by host tissues, whilst D-lactate is formed through bacterial fermentation in the large intestine. D-lactate, when formed in excess by bacteria in the colon, has been linked to gastrointestinal cancers; D-lactic acidosis can also potentially impact neurological diseases [21],[22]. The overall concentration of total lactate in the colon is largely dependent on the composition and abundance of lactate-producing and lactate-utilizing species. The overall net balance of these microbial species, in tandem with their metabolic outputs, which to some extent is driven by gut environmental conditions including pH levels, will therefore drive net total lactate levels.

## 3. Lactate-utilizing bacterial species from the human colon

Duncan et al. [23] isolated several species of lactate-utilizing bacteria, one of which was related to known species, such as *Eubacterium hallii* (now *Anaerobutyricum hallii*); this was the first report of their ability to use lactate. Other genera and species that can utilize lactate include *Anaerostipes caccae*, *Anaerostipes hadrus* [24], and *Anaerobutyricum soehngenii*, all of which utilize lactate to generate butyrate, which is considered the main fermentation product of these species. The biochemical pathways involved in this conversion suggest a stoichiometry of two moles of acetate and four moles of lactate being converted into three moles of butyrate. *Coprococcus catus* can also use lactate to form propionate by employing the acrylate pathway [25], as does *Megasphaera elsdenii*. *Veillonella* species metabolize lactate to propionate by employing the succinate pathway. Transcriptomic analysis of lactate-utilizing bacteria confirmed two gene clusters that are strongly up-regulated by lactate: *lct* in a butyrate-producing *A. soehngenii* strain and *lap* in a *C. catus* strain [26]. Lactate may also be utilized by several intestinal pathogens, including *Escherichia coli*, *Salmonella* species [27], and *Campylobacter jejuni* [28], which therefore makes the role of commensal gut anaerobes in competing with these pathogens extremely important to maintain healthy colonic function. For health maintenance, it is important to maintain a fine balance of intestinal bacterial species with the aim of controlling runaway lactic acidosis to prevent potential disease (as shown in Figure 1). Here, we investigated the potential of key lactate utilizers to form not only butyrate [17] but also propionate as a fermentation end product.

## 4. Genome mining of lactate utilizers

To illustrate the metabolic capabilities of the most representative lactate-utilizing bacteria, the genomic traits of 40 microbial strains reported by Sheridan et al. [26] were annotated. The selection of strains was based on those that have been reported to have the ability to metabolize lactate in the intestine, and several of which have remarkable lactate-utilizing abilities. These strains belong to the following species: *Anaerostipes hadrus* (n = 11), *Coprococcus catus* (n = 6), *Anaerobutyricum hallii* (n = 5), *Eubacterium limosum* (n = 3), *Anaerobutyricum soehngenii* (n = 2), *Anaerostipes caccae* (n = 2), *Anaerostipes* sp. (n = 1), *Anaerotignum propionicum* (n = 1), *Bacillus subtilis* (n = 1), *Campylobacter jejuni* (n = 1), *Coprococcus* sp. (n = 1), *Escherichia coli* (n = 1), *Megasphaera elsdenii* (n = 1), *Roseburia hominis* (n = 1), *Salmonella typhimurium* (n = 1), *Shewanella oneidensis* (n = 1), and *Veillonella parvula* (n = 1). Publicly available reference genome sequences were retrieved from the National Center for Biotechnology Information (NCBI) Assembly database (NCBI accession codes: GCA_000005845.2, GCA_000022165.1, GCA_000024945.1, GCA_000146165.2, GCA_000152245.2, GCA_000154245.1, GCA_000154305.1, GCA_000154545.1, GCA_000185825.1, GCA_000210555.1, GCA_000210695.1, GCA_000225345.1, GCA_000283495.2, GCA_000332875.2, GCA_000807675.2, GCA_000876135.1, GCA_001404655.1, GCA_001404835.1, GCA_001404955.1, GCA_001405175.1, GCA_001405635.1, GCA_001405715.1, GCA_001406155.1, GCA_001481725.1, GCA_001561955.1, GCA_002055965.1, GCA_003433855.1, GCA_003434235.1, GCA_017776255.1, GCA_017776275.1, GCA_017776335.1, GCA_017776345.1, GCA_017776395.1, GCA_017776445.1, GCA_017776475.1, GCA_018785175.1, GCA_018785195.1, GCA_900209925.1, GCA_900475265.1, and GCA_902381825.1).

### 4.1. Butyrate and propanediol pathway in lactate utilizers

The formation of butyrate by lactate-utilizing species and, in particular, the conversion of lactate to propanediol, are key processes in the growth and survival of certain gut anaerobes, being understudied in human colonic bacteria. To illustrate differences in the metabolic versatility and capabilities of lactate-utilizing bacteria, we investigated the presence of enzyme domains involved in butyrate formation, propanediol utilization, and the propanediol pathway for propionate production (Figure 2). For this purpose, butyrate and propanediol pathway enzymes were annotated in the genome sequences of representative lactate-utilizers [26] using HMMER v.3.4. This software implements profile hidden Markov models to map for searching sequence databases for sequence homologs. Propanediol pathway metabolic activities were annotated using Pfam and NCBIfam databases.

**Figure 2.**
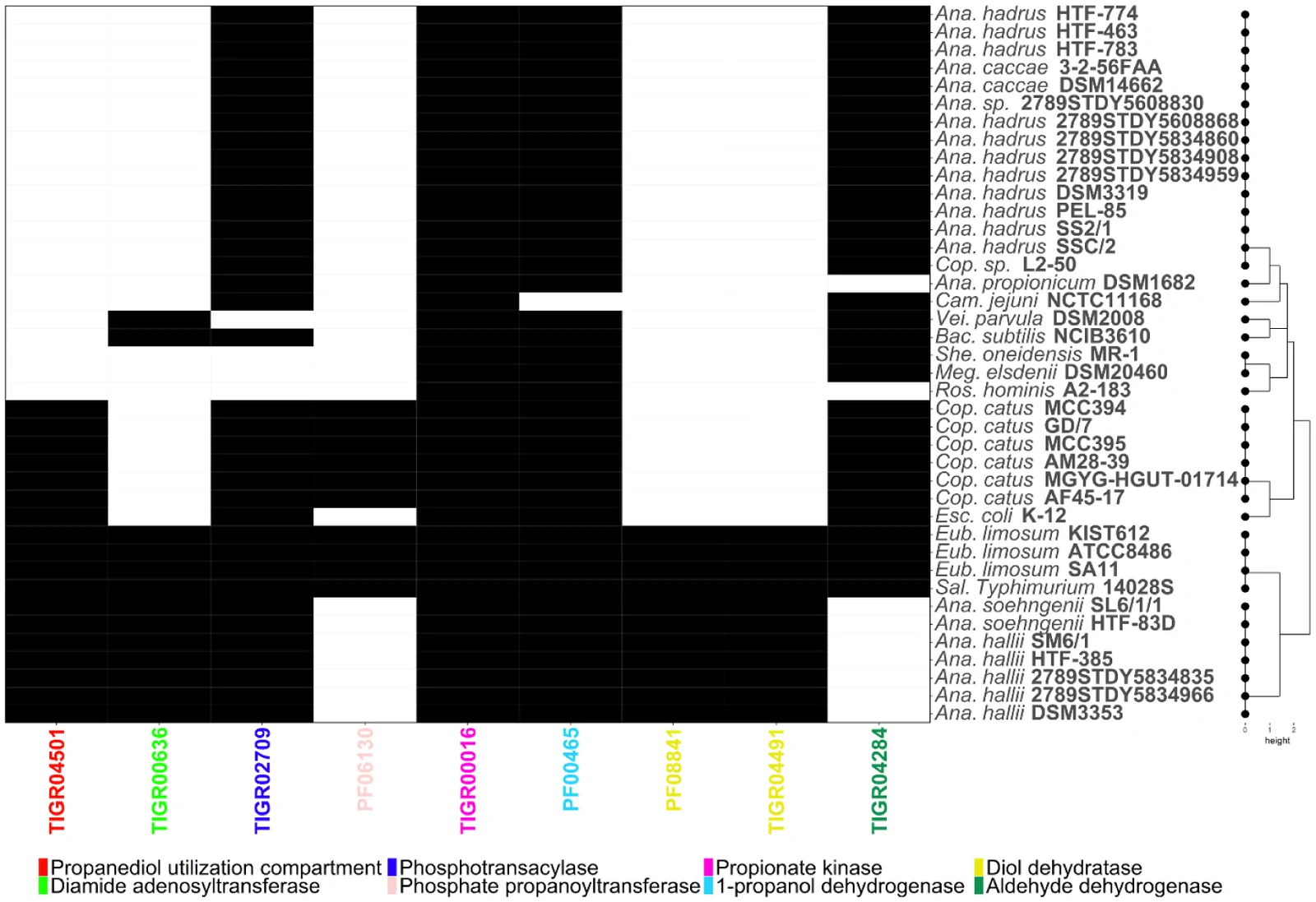
Identification of enzyme domains involved in propanediol utilization and the propanediol pathway for propionate production in lactate-utilizing bacterial strains. Protein domains characteristic of these enzymes were annotated in the genome sequences of lactate-utilizing bacteria using HMMER software and compared with the Pfam and NCBIfam databases. The presence or absence of each enzyme in the genome sequence of each bacterial strain is indicated by black or white cells respectively. The color scale on the x-axis indicates the type of enzyme involved in lactate metabolism. The enzyme profiles of these strains, grouped by similarity, are shown in a hierarchical cluster on the y-axis.

Genome analysis revealed that the butyryl CoA:acetate CoA-transferase route was the dominant pathway for butyrate formation, with the exception of *B. subtilis* NCBIB3610 and *C. jejuni* NCTC11168, both of which are not considered butyrate producers (data not shown). The propanediol utilization compartment was found in *A. halli*, *A. soehngenii*, *E. limosum*, and *C. catus*. These bacterial species also showed the widest range of enzymes involved in the propanediol pathway. Interestingly, the presence of 1,2-propanediol pathway enzymes in *A. halli* from fecal samples of human subjects has been previously reported [6]. On the other hand, phosphotransacylase, propionate kinase, and 1-propanol dehydrogenase enzymes were found in the majority of strains. Similarly, aldehyde dehydrogenase was determined in the genome sequences of all lactate utilizers with the exception of *A*. *halli*, *A*. *soehngenii*, *A*. *propionicum*, and *R*. *hominis* strains. In contrast, phosphate propanoyltransferase was exclusively found in *C*. *catus*, *E*. *limosum*, and *S. typhimurium*. Diamide adenosyltransferase was also annotated in the genome sequences of *A*. *halli*, *A*. *soehngenii*, *B*. *subtilis*, and *S*. *oneidensis*. Microbial diversity and metabolic plasticity are clearly important traits to help maintain healthy gut function.

### 4.2. Metabolic network and cross-feeding of lactate utilizers

To investigate the metabolic synergy between representative lactate-utilizing bacteria, microbial genomes of selected strains [26] were annotated using Prokka (v1.14.6) to generate standard Genbank files (.gbk) that were used as input for metage2metabo (v1.5.0) [29],[30]. This software allows the reconstruction of a genome-scale metabolic network (GSMN) of lactate utilizers. To simulate microbial metabolic interactions in the colon, a “seeds” file containing pectin and different nutrients that may be found in the human gut, according to previous studies, was provided [29],[30].

Metabolic network modeling revealed that most of the strains were butyrate producers. In addition, 10 lactate-utilizing strains, including *A. propionicum* DSM1682, *E. limosum* ATCC8486, *R. hominis* A2-183, *A. hadrus* HTF-463, *A. caccae* DSM14662, and *V. parvula* DSM2008, among others, are essential symbionts. In the model, these strains were crucial in obtaining all producible metabolites in the gut microbial community (Figure 3). These key microbes may establish synergistic interactions with other microbial groups, including other *Anaerostipes* (*A. caccae*, *A. hadrus*, *A. hallii*), *Coprococcus* (*C. catus*), and *Eubacterium* (*E. limosum*) strains. In addition, potential synergistic mechanisms between *S. typhimurium*, *S. oneidensis*, and several *C. catus* strains were determined. Similarly, *E. limosum* and *E. coli* K-12 may interact with *A. soehngenii* and *A. hallii* strains.

Metabolites produced by the microbial community through cross-feeding mechanisms include: i) oxo fatty acids and carboxy acids (oxopentenoate and 4-oxalomesaconate), ii) sugar derivatives [galactitol-1-phosphate (Gal-1P) and keto-D-tagatose 6-phosphate], iii) lipids [(2E)-2-hydroxypenta-2,4-dienoate and (2Z)-2-hydroxypenta-2,4-dienoate], and iv) amino acids and derivatives (sulfo-cysteine). The biological role of some of these metabolites has been previously reported. Oxopentanoate and other oxo-fatty acids may be produced from the breakdown of dietary unsaturated fatty acids by gut bacteria [31]. On the other hand, 4-oxalomesaconate [32] and 2-hydroxypenta-2,4-dienoate [33] are key intermediates in the degradation of aromatic compounds. Regarding carbohydrate metabolites, Gal-1P is a precursor to galactitol, which is a source of energy in bacteria [34], and keto-D-tagatose 6-phosphate is involved in the catabolism of lactose and galactose [35]. Finally, sulfo-cysteine is a precursor of cysteine that can be produced by certain bacteria [36].

The impact of lactate on function has been previously studied using *in vitro* model fermentor systems, whereby lactate was supplied continuously to human gut microbial communities using continuous culture at constant pH values of 5.5 and 6.5 (modeling the pH in the proximal and distal colon, respectively) [14]. From the metabolite (short-chain fatty acids) profiles and using 16S rRNA gene sequence analysis of the microbial communities, it was apparent that there was a major shift in the balance of the community, with Bacteroidetes and Bacillota (previously Firmicutes) being replaced with other phyla, including Actinobacteria and Proteobacteria. This coincided with altered lactate utilization and metabolite output [14].

**Figure 3.**
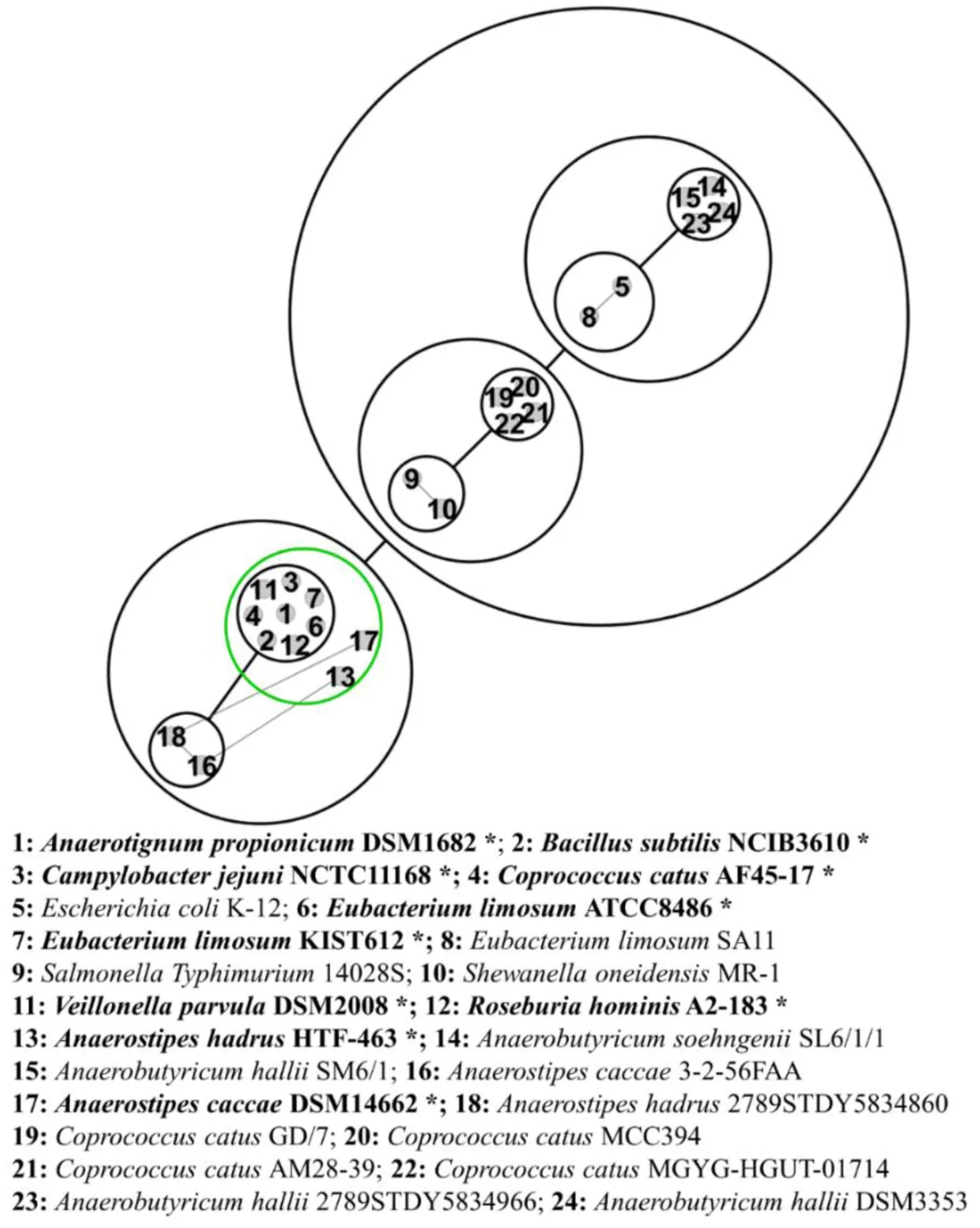
Metabolic network illustrating potential cross-feeding mechanisms between lactate-utilizing bacteria. The network nodes (i.e., circles containing different microbial communities showing equivalent metabolic functions) are connected by black lines and indicate synergistic relationships between communities and complementary metabolic functions. The green node corresponds to essential symbionts (i.e., key species needed to obtain all producible metabolites in the gut microbial community, highlighted in bold, *). It should be noted that microbes inside the same node (black circle) play a similar metabolic role and could be replaced by other members from the same community, with the exception of essential symbionts included in the green node.

### 4.3. Mining for antibiotic resistance genes in lactate-utilizing bacteria

Antibiotic-resistant bacteria in the human colon are a major health concern, as the overuse of antibiotics in medicine and agriculture has accelerated the enrichment of resistant bacteria in the gut [37]. The colon acts as a reservoir for antibiotic-resistant bacteria that can spread through horizontal gene transfer within the wider microbial community [38]. When considering the development of new probiotic strains, particularly those derived from the gut, it is crucial that these do not carry multi-antibiotic resistance traits [39]. Therefore, genomes were also mined for the presence of antibiotic resistance genes, which were absent in representative genomes, suggesting that these strains would be excellent biotherapeutic candidates. To assess the safety of lactate-utilizing bacteria use, antibiotic resistance genes (ARGs) were annotated in the genome sequences of representative lactate-utilizers [26] using the ABRicate software and ResFinder database [40]. As can be seen in Figure 4, several ARGs were identified in some lactate-utilizing strains. In this regard, the widest range of ARGs conferring resistance to aminoglycosides, beta-lactams, macrolides, or tetracyclines was found in the sequences of *C. catus* AM28-39 and *Roseburia hominis* A2-183. However, no multidrug resistance strains were identified in *Anaerostipes* species, *Coprococus catus*, *Eubacterium limosum*, *Megasphaera elsdenii*, and *Veillonella parvula* (Figure 4).

**Figure 4.**
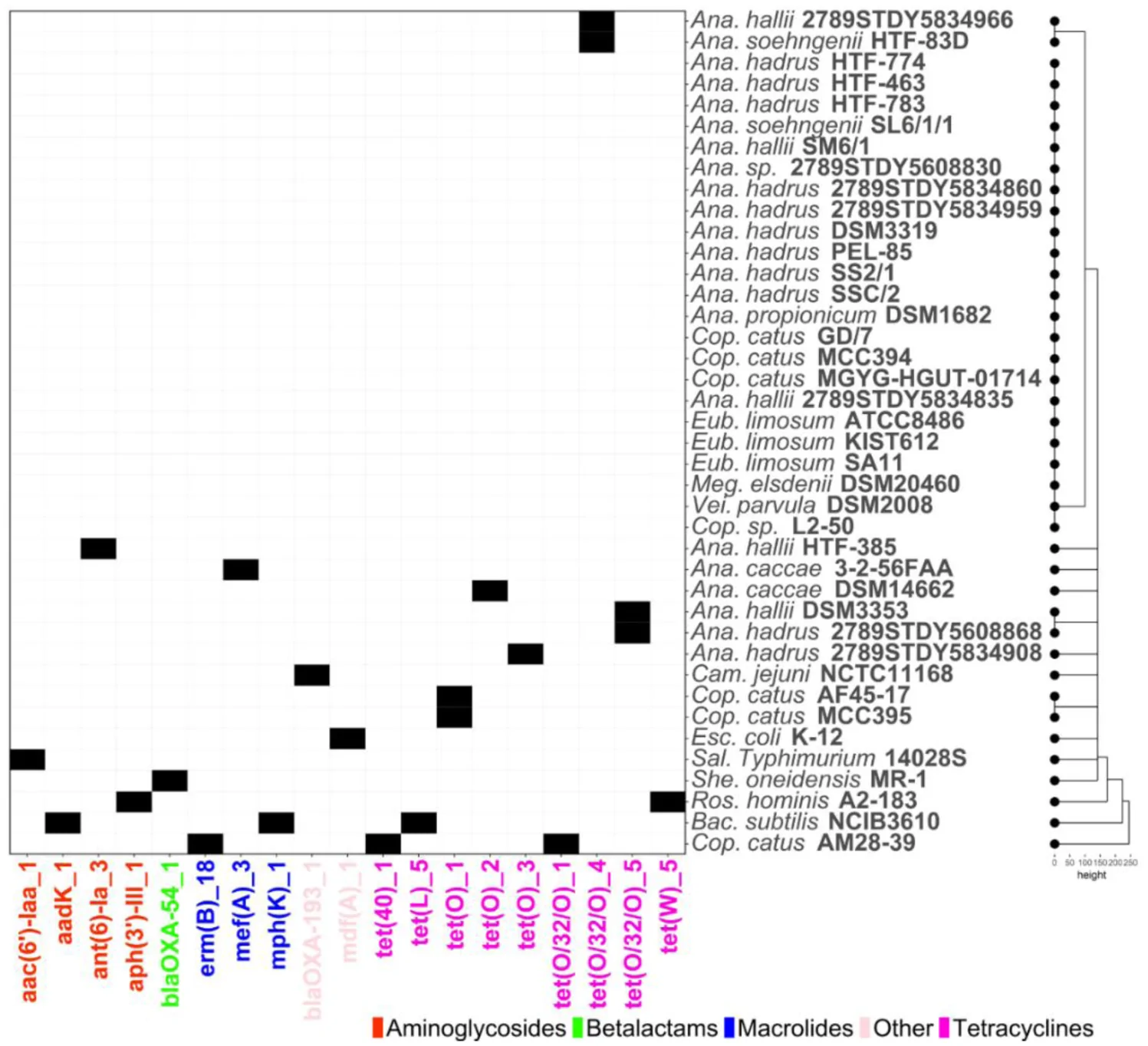
Identification of antimicrobial resistance genes (ARGs) in lactate-utilizing bacteria. The genome sequences of lactate-utilizing bacteria were analyzed using ABRicate software and ResFinder database [40] to identify genes associated with resistance to antimicrobials. Genome sequences were retrieved from the National Center for Biotechnology Information (NCBI) repository. The presence or absence of each ARG in the genome sequence of each bacterial strain is indicated by black or white cells. The color scale on the x-axis indicates the type of antimicrobial resistance that is associated with each gene. The ARG profiles of these strains, grouped by similarity, are shown in a hierarchical cluster on the y-axis.

## 5. Clinical applications

In the healthy colon, lactate normally remains at low-to-undetectable levels. Under pathological conditions, however, excessive lactate production or reduced lactate utilization can result in increased lactate accumulation, which has been associated with several disease states. For example, there is an evident association between lactate metabolism and inflammatory bowel disease (IBD) in patients often exhibiting reduced numbers of lactate-utilizing bacteria and increased concentrations of intestinal total lactate, resulting in lactic acidosis. The proposed mechanisms include loss of butyrate, thereby reducing energy supply to the colonocytes, as it has been estimated that up to 20% of butyrate is derived from lactate metabolism [41]. Butyrate, along with acetate and propionate, is an important signaling molecule that influences both innate and adaptive immune responses. Lactate accumulation lowers intestinal pH and may inhibit the growth of certain gut anaerobes. Moreover, the disruption in community structure may favor the growth of D-lactate-producing bacteria and opportunistic pathogens. Therefore, there is keen interest in using next-generation probiotics or biotherapeutics to promote health, and lactate-utilizing bacterial species are of considerable interest as potential therapeutic probiotics that could help to prevent microbial and metabolic imbalance [39]. Depending on parameters such as pH, lactate supply could encourage the metabolism of lactate by lactate-utilizing populations of bacteria and help to prevent lactate accumulation, resulting in increased butyrate and propionate formation [42]. Below a certain threshold, however, at low pH values, this is likely to destabilize microbial communities, impacting colonization stability and microbial competition, with resultant lactate accumulation [14]. The development of new probiotic therapeutics will, however, require thorough investigation of potential microbes at the strain level. It is crucial that these bacterial strains do not carry multi-antibiotic resistance traits and are generally regarded as safe to use.

This may, however, be reversed using next-generation probiotics. In particular, several studies have now been conducted with the *A. soehngenii* strain L2-7 [43]. Initial studies, conducted with the wild-type strain L2-7 in small animals, showed increased butyrate levels and improvements in insulin sensitivity compared to controls [44]. Both *in vitro* and small-animal studies have therefore led to interest in human clinical studies.

There are clearly alternative approaches also being considered to modulate gut microbiota, such as fecal microbiota transplantation to support healthy gut function. There may, however, be potentially serious risks associated with this strategy, such as poorly examined samples leading to infections and the introduction of antibiotic resistance genes [45]. Single-strain or strain combinations that are very well-characterized may offer considerable advantages, including developing lactate-utilizing, butyrate-producing bacteria to promote health. For example, previous studies showed that co-culturing *A. soehngenii* (previously *E. hallii*) with a lactate-producing bacterium (*B. adolescentis*) growing on starch resulted in efficient lactate cross-feeding to form butyrate [23].

Moreover, it is important that strains used as next-generation probiotics or biotherapeutics are free of antibiotic resistance traits and generally regarded as safe to use. Interestingly, the majority of lactate-utilizing *A. hadrus*, *C. catus*, and *E. limosum* strains showed no ARGs (Figure 4). Despite interest in using *A. soehngenii* strain L2-7 (*A. hallii* DSM3353) as a therapeutic, this wild-type strain carries tetracycline-resistance genes. Exposure of cells of this strain to ethyl methane sulfonate, however, resulted in the selection of a tetracycline-sensitive derivative, strain CH-106, which in other respects was virtually the same as the parent strain [46], increasing its usefulness as a therapeutic.

*Anaerobutyricum soehngenii* clearly shows promise in promoting health, as a short-term human intervention trial administering *A. soehngenii* in 24 subjects with metabolic syndrome showed improvements in insulin sensitivity after four weeks [47]. A longer-term study using encapsulated cells in a larger cohort of adults also showed improved glycemic control. Moreover, diastolic blood pressure was significantly reduced in the treated subjects [48], indicating the usefulness of this therapeutic approach to improve cardiometabolic health of prediabetic adults. Collectively, these studies provide a robust evidence base for the rational design of precision microbiota-targeted interventions to modulate gut and systemic health.

There are clearly key human gut bacterial species that represent a largely untapped reservoir of microbes with immense potential to be developed as next-generation probiotics [39]. Here, we focused on lactate-utilizing bacterial species, including *Anaerobutyricum soehngenii*, that are urgently awaiting development as new biotherapeutics to advance human health, which could be supported by machine learning algorithms [11],[49].

## 6. Conclusions

Numerous studies support the development of precision microbiota-based interventions for gut and systemic health. Genome mining of key human colonic lactate-utilizing bacterial species has identified several strains that, in addition to being known butyrate producers, also have the potential to generate propionate using the propanediol pathway. The ability to utilize lactate largely tends to be a species-dependent phenomenon, and metabolic modeling highlights the role of key microbes, including *Anaerobutyricum*, *Anaerostipes*, *Anaerotignum*, *Eubacterium*, and *Roseburia* strains, that may establish cross-feeding interactions with other lactate utilizers to maximize metabolite production. Unlike lactate utilization, which generally occurs at the bacterial species level, antibiotic resistance is a strain-dependent phenomenon, and further genome mining identified strains with no antibiotic resistance genes. These bacterial species, which need to be assessed at the strain level for translational relevance, therefore represent microbes with potential to be developed as new biotherapeutics.

## Use of AI tools declaration

The authors declare they have not used Artificial Intelligence (AI) tools in the creation of this article.
